# Insulin Regulates AKT/GSK-3β Signalling, Tau Phosphorylation, and Redox Homeostasis in SH-SY5Y Neuroblastoma Cells

**DOI:** 10.3390/ijms27125565

**Published:** 2026-06-19

**Authors:** Adrian Jorda, Kenia Alvarez-Gamez, Sara Vergani, Ilenia Paba, Mar Perez, Martin Aldasoro, Jose M. Vila, Soraya L. Valles

**Affiliations:** 1 Department of Physiology, School of Medicine, University of Valencia, Blasco Ibañez 15, 46010 Valencia, Spain; adrian.jorda@uv.es (A.J.); ileniapaba@gmail.com (I.P.); martin.aldasoro@uv.es (M.A.);; 2 Department of Nursing, Faculty of Nursing and Podiatry, University of Valencia, Menéndez y Pelayo 19, 46010 Valencia, Spain

**Keywords:** insulin, AKT, GSK-3β, NRF2, TAU, oxidative stress, SH-SY5Y cells

## Abstract

Insulin (Ins) regulates multiple intracellular signalling pathways involved in cell survival, oxidative stress responses, and tau phosphorylation. Dysregulation of these pathways has been implicated in neurodegenerative disorders, including Alzheimer’s disease (AD). The present study evaluated the effects of insulin on protein kinase B/glycogen synthase kinase-3 beta (AKT/GSK-3β) signalling, tau phosphorylation, and oxidative stress-related markers in SH-SY5Y neuroblastoma cells. Cell metabolic activity was assessed using the (diphenyltetrazolium bromide) MTT assay, while cell number and viability were evaluated by Trypan Blue exclusion, necrosis by lactate dehydrogenase (LDH) release, and apoptosis by Caspase-3 activity. Western blot analysis was performed to evaluate the expression of phosphorylated AKT (p-AKT), phosphorylated GSK-3β (p-GSK-3β Ser9), phosphorylated TAU (pTAU), nuclear factor erythroid 2-related factor 2 (NRF2), manganese superoxide dismutase (Mn-SOD), and copper/zinc superoxide dismutase (Cu/Zn-SOD). Lipid peroxidation was determined by measuring malondialdehyde (MDA) levels using a colorimetric/fluorometric assay. Insulin treatment increased MTT reduction (31.25%) and cell metabolic activity (119.15%) while reducing LDH release (19.2%) and Caspase-3 activity (31.26%). In addition, insulin significantly increased p-AKT (34.2%) and p-GSK-3β (Ser9) (19.9%) levels. A reduction in pTAU levels (53.39%) was also observed following insulin treatment. Furthermore, insulin increased NRF2 expression (18.77%), Cu/Zn-SOD (37.29%), and Mn-SOD (50.16%) and reduced MDA levels (13.95%). These findings indicate that insulin modulates signalling pathways associated with tau phosphorylation and cellular redox regulation in SH-SY5Y cells. Insulin treatment was associated with increased AKT and GSK-3β phosphorylation, reduced tau phosphorylation, and changes in oxidative stress-related markers in SH-SY5Y neuroblastoma cells. These findings support a role for insulin in the modulation of molecular pathways implicated in cellular stress responses and tau regulation. Further studies using differentiated neuronal models and disease-relevant conditions are required to determine the relevance of these observations to neurodegenerative disorders.

## 1. Introduction

Insulin (Ins) is increasingly recognized as an important regulator of central nervous system function beyond its classical role in peripheral glucose homeostasis. Insulin receptors are widely distributed throughout the brain, particularly in regions involved in learning, memory, and cognition, including the hippocampus, entorhinal cortex, and frontal cortex [1,2,3]. Through the activation of intracellular signalling cascades, insulin contributes to the regulation of synaptic plasticity, neuronal survival, energy metabolism, and cellular stress responses [4]. Alterations in brain insulin signalling have been associated with ageing and several neurodegenerative disorders, including Alzheimer’s disease (AD) [5,6,7,8,9,10].

One of the principal signalling pathways activated by insulin is the phosphoinositide 3-kinase (PI3K)/protein kinase B (AKT) pathway [11]. The activation of AKT promotes cell survival and regulates multiple downstream targets, including glycogen synthase kinase-3β (GSK-3β). The phosphorylation of GSK-3β at Ser9 inhibits its kinase activity and influences a variety of cellular processes, including metabolism, cell metabolic activity, apoptosis, and cytoskeletal regulation [12,13,14,15]. The dysregulation of the AKT/GSK-3β signalling axis has been reported in several pathological conditions and has attracted particular attention because of its potential involvement in neurodegenerative disorders [16,17,18].

Tau is a microtubule-associated protein that contributes to cytoskeletal organization and intracellular transport. The biological activity of tau is regulated by phosphorylation at multiple serine and threonine residues. Under physiological conditions, phosphorylation modulates tau function; however, excessive phosphorylation can alter its interaction with microtubules and has been associated with pathological processes in several tauopathies, including AD [19,20,21]. GSK-3β is one of the major kinases involved in tau phosphorylation and can target multiple phosphorylation sites within the protein [22,23]. Consequently, signalling pathways that regulate GSK-3β activity may influence tau phosphorylation status.

In addition to alterations in kinase signalling, oxidative stress is considered an important contributor to cellular dysfunction in neurodegenerative diseases. The transcription factor nuclear factor erythroid 2-related factor 2 (NRF2) plays a central role in the cellular antioxidant response by regulating the expression of genes involved in redox homeostasis, detoxification, and cytoprotection [23,24,25]. Under conditions of oxidative stress, NRF2 promotes the expression of antioxidant enzymes, including superoxide dismutase (SODs), catalase, and glutathione-related enzymes, thereby limiting oxidative damage and supporting cellular adaptation to stress [26,27]. Evidence suggests that interactions between NRF2 and GSK-3β may represent an important regulatory mechanism linking cellular signalling pathways with antioxidant defence systems [28,29].

SH-SY5Y cells are a human neuroblastoma-derived cell line widely used to investigate intracellular signalling pathways relevant to neuronal biology. Although undifferentiated SH-SY5Y cells do not fully reproduce the phenotype of mature neurons, they provide a useful experimental model for examining molecular responses to pharmacological and metabolic stimuli under controlled conditions [30,31].

The present study aimed to evaluate the effects of insulin on cell survival-related parameters, AKT/GSK-3β signalling, tau phosphorylation, and oxidative stress-associated markers in SH-SY5Y neuroblastoma cells. Specifically, we analyzed cell metabolic activity, necrosis, apoptosis, the expression of phosphorylated AKT, phosphorylated GSK-3β, phosphorylated tau, NRF2, Cu/Zn-superoxide dismutase (Cu/Zn-SOD), Mn-superoxide dismutase (Mn-SOD), and malondialdehyde (MDA) levels as an indicator of lipid peroxidation.

## 2. Results

### 2.1. Cell Metabolic Activity

**Figure 1:**
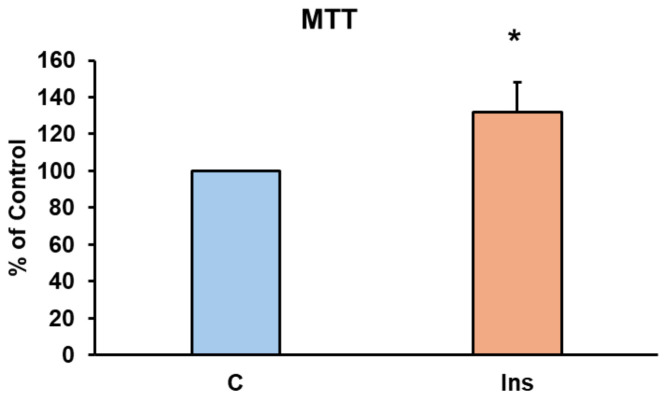
Effect of insulin on SH-SY5Y cells. Cell metabolic activity was determined by 3-(4,5-dimethylthiazol-2-yl)-2,5-diphenyltetrazolium bromide (MTT) assay in cells treated for 24 h in absence (control, C) or presence of insulin (10^−8^ M). Data are mean ± SD of independent experiments (*n* = 5). Data are expressed as percentage of control (100%). Statistical analysis was performed using Student’s *t*-test (* *p* < 0.05 vs. control).

The effect of insulin on SH-SY5Y cell metabolic activity was evaluated using the 3-(4,5-dimethylthiazol-2-yl)-2,5-diphenyltetrazolium bromide (MTT) reduction assay. As shown in Figure 1, treatment with insulin (10^−8^ M, 24 h) produced a significant increase in MTT reduction compared with control cells (31.25% increase vs. control). These results reflect changes in cellular metabolic activity associated with NADPH-dependent oxidoreductase activity and do not directly represent cell proliferation.

### 2.2. Effect of Insulin on SH-SY5Y Cell Number and Viability Assessed by Trypan Blue Exclusion

Cell number and viability were assessed using the Trypan Blue exclusion assay. This method reflects membrane integrity and allows for the determination of viable cell numbers. SH-SY5Y cells were treated with insulin (10^−8^ M) for 24 h. As shown in Table 1, insulin increased the viable cell number to 119.15% of control values.

### 2.3. Caspase-3 Activity

**Figure 2:**
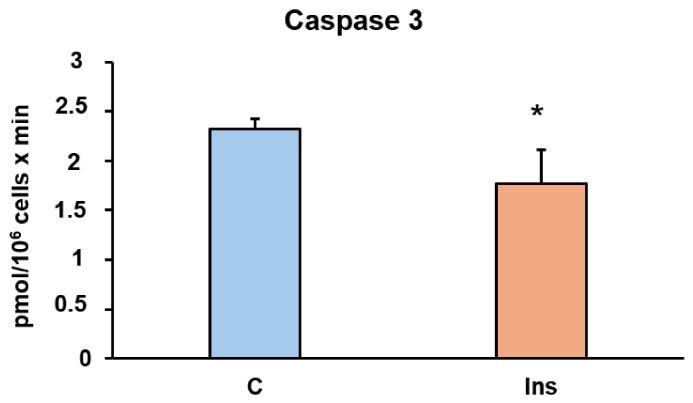
Caspase-3 activity in SH-SY5Y cells treated with insulin (10^−8^ M, 24 h). Data are mean ± SD of four independent experiments. Statistical analysis was performed using Student’s *t*-test (* *p* < 0.05 vs. control).

To evaluate apoptosis-related signalling, Caspase-3 activity was measured following insulin treatment (10^−8^ M, 24 h). As shown in Figure 2, insulin significantly reduced Caspase-3 activity compared with control cells (31.26% decrease vs. control). These results indicate that insulin modulates apoptosis-associated signalling pathways in SH-SY5Y cells under these experimental conditions.

### 2.4. Lactate Dehydrogenase (LDH) Release

**Figure 3:**
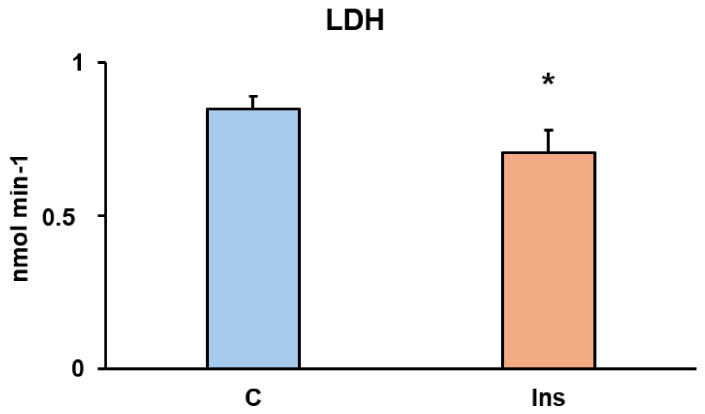
Lactate dehydrogenase (LDH) activity in SH-SY5Y cell culture supernatants after insulin treatment (10^−8^ M, 24 h). Data are mean ± SD of four independent experiments. Statistical analysis was performed using Student’s *t*-test (* *p* < 0.05 vs. control).

Cell membrane integrity was assessed by measuring LDH release into the culture medium. Insulin treatment (10^−8^ M, 24 h) significantly reduced LDH activity compared with control cells (0.70 ± 0.07 vs. 0.85 ± 0.04 nmol·min^−1^), corresponding to a 19.2% decrease. These results indicate reduced cytotoxicity under insulin-treated conditions in SH-SY5Y cells (Figure 3).

### 2.5. Protein Kinase B (AKT) Phosphorylation

**Figure 4:**
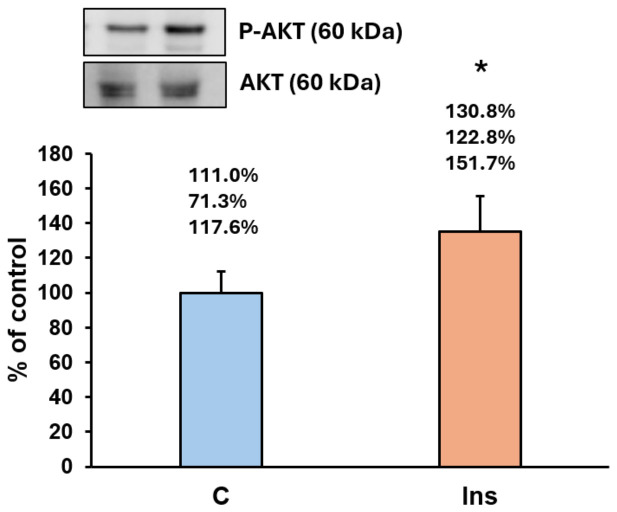
Protein kinase B (AKT) and p-AKT protein expression in SH-SY5Y cells after 30 min insulin treatment (10^−8^ M). Representative Western blots of p-AKT and total AKT are shown. Densitometric quantification of p-AKT was normalized to total AKT and expressed as percentage of control. Each point represents independent biological replicate (*n* = 4 per group). Data are presented as mean ± SD. Statistical analysis was performed using unpaired two-tailed Student’s *t*-test (* *p* < 0.05 vs. control). Individual data points are shown in quantification plot to illustrate biological variability.

Insulin treatment (10^−8^ M, 30 min) increased AKT phosphorylation in SH-SY5Y cells compared with the control. Densitometric analysis of p-AKT normalized to total AKT was expressed as a percentage of the control. Insulin significantly increased p-AKT levels compared with the control. Individual biological replicates showed values ranging from 128.0% to 158.3% in the insulin group, whereas control values ranged from 74.4% to 122.8%. Overall, insulin treatment resulted in a significant increase in AKT phosphorylation (approximately 45%) compared with the control (Figure 4). Individual data points representing independent biological replicates are shown in the quantification plot.

### 2.6. Glycogen Synthase Kinase-3 Beta (GSK-3β) Phosphorylation

**Figure 5:**
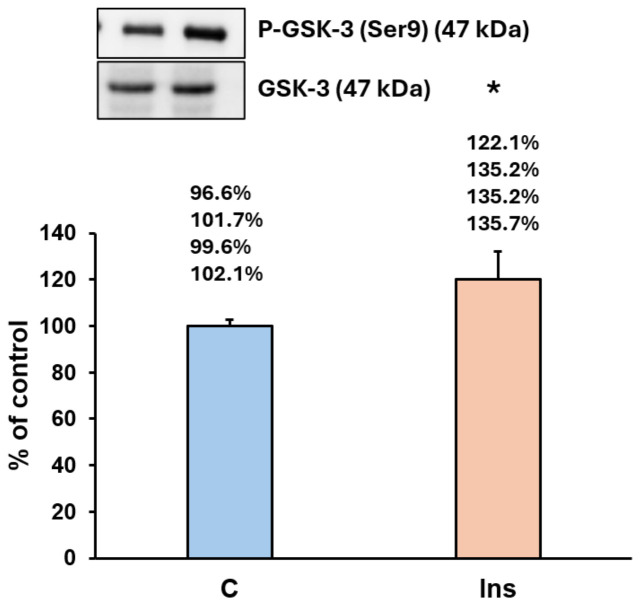
Glycogen synthase kinase-3 beta GSK-3 and p-GSK-3β (Ser9) protein expression in SH-SY5Y cells after 30 min insulin treatment (10^−8^ M). Representative Western blots of p-GSK-3β (Ser9) and total GSK-3 are shown. Densitometric quantification of p-GSK-3β (Ser9) was normalized to total GSK-3 and expressed as percentage of control. Each point represents independent biological replicate (*n* = 4 per group). Data are presented as mean ± SD. Statistical analysis was performed using unpaired two-tailed Student’s *t*-test (* *p* < 0.05 vs. control). Individual data points are shown in quantification plot to illustrate biological variability.

Insulin treatment (10^−8^ M, 30 min) significantly increased GSK-3 phosphorylation at Ser9 in SH-SY5Y cells compared with the control. Densitometric analysis of p-GSK-3β (Ser9) normalized to total GSK-3 was expressed as a percentage of the control. Insulin induced a robust increase in phosphorylation levels compared with control cells. Individual biological replicates showed values ranging from 122.1% to 135.7% in the insulin group, whereas control values ranged from 96.6% to 102.1%. Overall, insulin treatment resulted in a significant increase in GSK-3 phosphorylation (approximately 32%) compared with the control (Figure 5). Individual data points representing independent biological replicates are shown in the quantification plot.

### 2.7. TAU Phosphorylation

**Figure 6:**
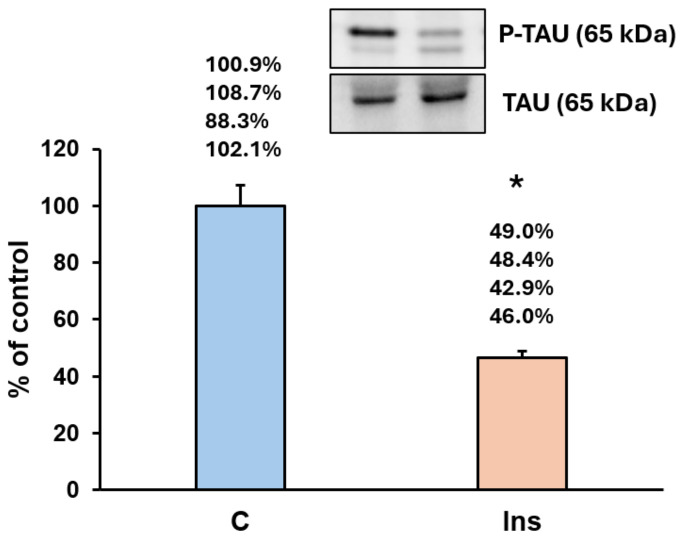
Phospho-TAU (Ser396) and total TAU protein expression in SH-SY5Y cells after insulin treatment (10^−8^ M, 30 min). Representative Western blots of phospho-TAU (Ser396) and total TAU are shown. Densitometric quantification of phospho-TAU (Ser396) normalized to total TAU was expressed as percentage of control. Each point represents independent biological replicate (*n* = 4 per group). Data are presented as mean ± SD. Statistical analysis was performed using unpaired two-tailed Student’s *t*-test (* *p* < 0.05 vs. control). Individual data points are shown in quantification plot to illustrate biological variability.

Insulin treatment (10^−8^ M, 30 min) significantly reduced phospho-TAU (Ser396) levels in SH-SY5Y cells compared with the control. Densitometric analysis of phospho-TAU (Ser396) normalized to total TAU was expressed as a percentage of the control. Insulin induced a marked decrease in TAU phosphorylation. Individual biological replicates showed values ranging from 42.9% to 49.0% in the insulin group, whereas control values ranged from 88.3% to 108.7%. Overall, insulin treatment resulted in an approximate 50% reduction in phospho-TAU (Ser396) levels compared with the control (Figure 6). Individual data points representing independent biological replicates are shown in the quantification plot.

### 2.8. Nuclear Factor Erythroid 2-Related Factor 2 (NRF2) Expression

**Figure 7:**
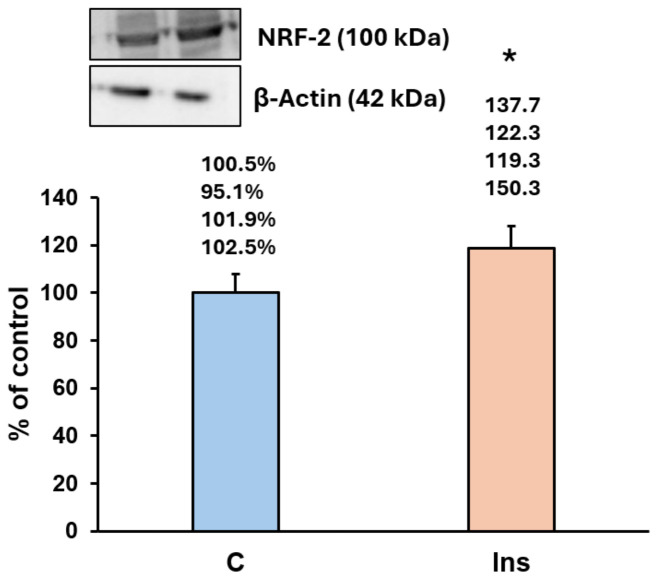
Nuclear factor erythroid 2-related factor 2 (NRF-2) and β-actin protein expression in SH-SY5Y cells after insulin treatment (10^−8^ M, 30 min). Representative Western blots of NRF-2 and β-actin are shown. Densitometric quantification of NRF-2 normalized to β-actin was expressed as percentage of control. Each point represents independent biological replicate (*n* = 4 per group). Data are presented as mean ± SD. Statistical analysis was performed using unpaired two-tailed Student’s *t*-test (* *p* < 0.05 vs. control). Individual data points are shown in quantification plot to illustrate biological variability.

Insulin treatment (10^−8^ M, 30 min) significantly increased NRF-2 protein levels in SH-SY5Y cells compared with the control. Densitometric analysis of NRF-2 normalized to β-actin was expressed as a percentage of the control. Insulin induced an increase in NRF-2 expression relative to control cells. Individual biological replicates showed values ranging from 119.3% to 150.3% in the insulin group, whereas control values ranged from 95.1% to 102.5%. Overall, insulin treatment resulted in an approximate 30–40% increase in NRF-2 levels compared with the control (Figure 7). Individual data points representing independent biological replicates are shown in the quantification plot.

### 2.9. Lipid Peroxidation, Malondialdehyde (MDA) Levels

**Figure 8:**
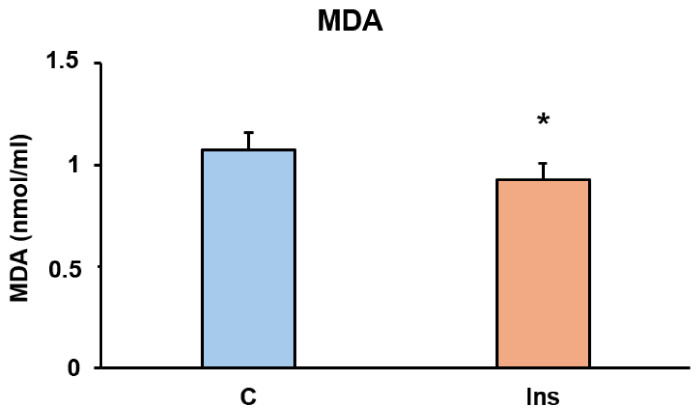
Malondialdehyde (MDA) levels in SH-SY5Y cell culture supernatants after insulin treatment (1 h). Data are mean ± SD of independent experiments (*n* = 4). Statistical analysis was performed using Student’s *t*-test (* *p* < 0.05 vs. control).

Lipid peroxidation was assessed by measuring MDA levels in culture supernatants. Insulin treatment (10^−8^ M, 1 h) reduced MDA levels from 1.075 to 0.925 nmol/mL, corresponding to a 13.95% decrease compared with control cells. These results indicate reduced lipid peroxidation under insulin-treated conditions (Figure 8).

### 2.10. Copper/Zinc Superoxide Dismutase (Cu/Zn-SOD) Expression

**Figure 9:**
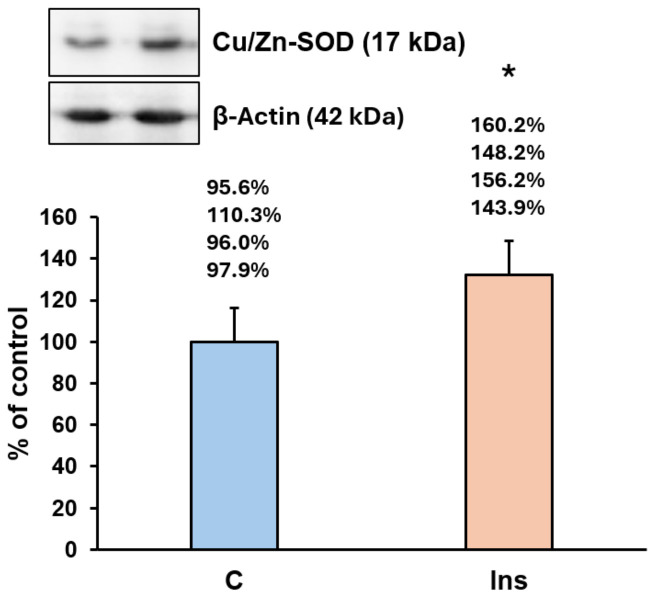
Copper/zinc superoxide dismutase (Cu/Zn-SOD) and β-actin protein expression in SH-SY5Y cells after insulin treatment (10^−8^ M, 30 min). Representative Western blots of Cu/Zn-SOD and β-actin are shown. Densitometric quantification of Cu/Zn-SOD normalized to β-actin was expressed as percentage of control. Each point represents independent biological replicate (*n* = 4 per group). Data are presented as mean ± SD. Statistical analysis was performed using unpaired two-tailed Student’s *t*-test (* *p* < 0.05 vs. control). Individual data points are shown in quantification plot to illustrate biological variability.

Insulin treatment (10^−8^ M, 30 min) significantly increased Cu/Zn-SOD protein expression in SH-SY5Y cells compared with the control. Densitometric analysis of Cu/Zn-SOD normalized to β-actin was expressed as a percentage of the control. Insulin induced a marked increase in Cu/Zn-SOD levels relative to control cells. Individual biological replicates showed values ranging from 143.9% to 160.2% in the insulin group, whereas control values ranged from 95.6% to 110.3%. Overall, insulin treatment resulted in an approximate 50–60% increase in Cu/Zn-SOD expression compared with the control (Figure 9). Individual data points representing independent biological replicates are shown in the quantification plot.

### 2.11. Manganese Superoxide Dismutase (Mn-SOD) Expression

**Figure 10:**
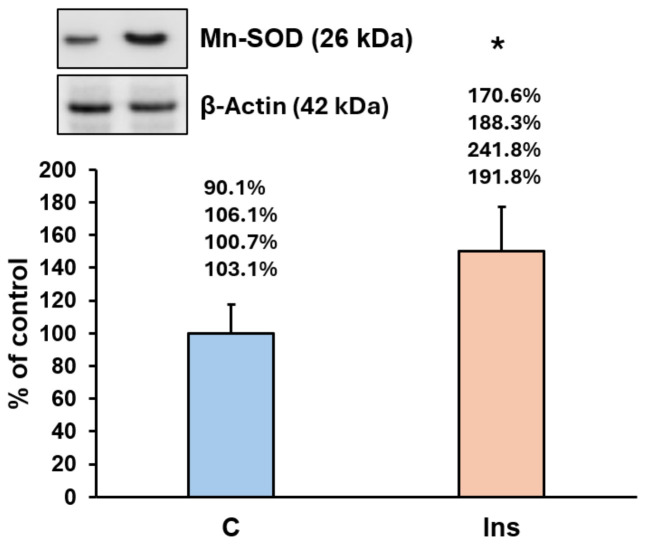
Manganese superoxide dismutase (Mn-SOD) and β-actin protein expression in SH-SY5Y cells after insulin treatment (10^−8^ M, 30 min). Representative Western blots of Mn-SOD and β-actin are shown. Densitometric quantification of Mn-SOD normalized to β-actin was expressed as percentage of control. Each point represents independent biological replicate (*n* = 4 per group). Data are presented as mean ± SD. Statistical analysis was performed using unpaired two-tailed Student’s *t*-test (* *p* < 0.05 vs. control). Individual data points are shown in quantification plot to illustrate biological variability.

Insulin treatment (10^−8^ M, 30 min) significantly increased Mn-SOD protein expression in SH-SY5Y cells compared with the control. Densitometric analysis of Mn-SOD normalized to β-actin was expressed as a percentage of the control. Insulin induced a marked increase in Mn-SOD levels relative to control cells. Individual biological replicates showed values ranging from 170.6% to 241.8% in the insulin group, whereas control values ranged from 90.1% to 106.1%. Overall, insulin treatment resulted in an approximate 80–100% increase in Mn-SOD expression compared with the control (Figure 10). Individual data points representing independent biological replicates are shown in the quantification plot.

## 3. Discussion

In the present study performed in SH-SY5Y neuroblastoma cells, insulin modulated cell survival-related parameters, increasing cell metabolic activity and cell number while reducing markers associated with cell death, including Caspase-3 activity and LDH release. In parallel, insulin activated the PI3K/AKT signalling pathway, as evidenced by increased AKT phosphorylation, and it increased the inhibitory phosphorylation of GSK-3β at Ser9. In addition, insulin reduced the phosphorylation of TAU at Ser396 and was associated with increased NRF2 protein expression, together with changes in oxidative stress-related markers, including decreased MDA levels and increased Cu/Zn-SOD and Mn-SOD expression.

The observed effects of insulin on cell metabolic activity and cell number are consistent with previous reports describing insulin as a modulator of growth and survival signalling in SH-SY5Y cells and other cellular models [32,33]. The MTT assay reflects cellular metabolic activity rather than direct proliferation, as it depends on mitochondrial and cytosolic oxidoreductase activity. Insulin is a well-established activator of the PI3K/AKT pathway, which plays a central role in regulating cell survival, metabolism, and stress responses [34,35,36,37]. The activation of AKT leads to the phosphorylation and inhibition of downstream targets such as GSK-3β, thereby modulating multiple intracellular processes [38,39]. In the present study, the insulin-induced reduction in Caspase-3 activity and LDH release is consistent with the modulation of apoptosis- and membrane integrity-related pathways downstream of AKT signalling. However, these effects should be interpreted as changes in cell death-related markers rather than direct evidence of neuroprotection, given the use of a neuroblastoma-derived cell line [40,41].

GSK-3β is a constitutively active kinase involved in multiple cellular functions, including metabolism, proliferation, and cytoskeletal regulation [31,32]. The inhibition of GSK-3β through phosphorylation at Ser9 by AKT reduces its kinase activity and modulates downstream signalling pathways [42,43]. One of the best-characterized substrates of GSK-3β is TAU protein, which is involved in microtubule stability and intracellular organization [43,44]. The hyperphosphorylation of TAU reduces its affinity for microtubules and is a biochemical feature associated with tauopathies, including Alzheimer’s disease [45,46]. GSK-3β has been identified as one of the major kinases involved in TAU phosphorylation at multiple sites, including Ser396 [42,43]. In this study, insulin was associated with increased inhibitory phosphorylation of GSK-3β and reduced phospho-TAU (Ser396) levels, suggesting the modulation of signalling pathways regulating TAU phosphorylation in SH-SY5Y cells. However, given the absence of a disease-inducing condition, these findings should be interpreted as the regulation of TAU phosphorylation under basal experimental conditions rather than as evidence of the prevention of neurodegenerative processes [47,48,49].

NRF2 is a transcription factor that regulates cellular antioxidant and stress response pathways [50,51]. Under conditions of oxidative stress, NRF2 controls the expression of genes involved in redox homeostasis, including antioxidant enzymes and detoxification systems [52,53]. In the present study, insulin increased NRF2 protein expression in SH-SY5Y cells, together with changes in oxidative stress-related markers. However, NRF2 activation was not directly assessed, as nuclear translocation or transcriptional activity of NRF2 target genes was not measured. Therefore, the observed increase in NRF2 should be interpreted as changes in protein expression rather than confirmed pathway activation.

The NRF2 and GSK-3β pathways are functionally interconnected. GSK-3β has been reported to negatively regulate NRF2 stability and activity, whereas the inhibition of GSK-3β may facilitate NRF2-mediated antioxidant responses [54,55]. In this context, the insulin-induced activation of AKT and subsequent inhibitory phosphorylation of GSK-3β may contribute indirectly to the modulation of NRF2-associated responses observed in this study. Consistent with this interpretation, insulin treatment was associated with reduced lipid peroxidation, as indicated by decreased MDA levels and increased expression of antioxidant enzymes Cu/Zn-SOD and Mn-SOD. These enzymes play complementary roles in cellular redox homeostasis, with Mn-SOD localized in mitochondria and Cu/Zn-SOD in the cytosol [56,57,58,59].

Oxidative stress and mitochondrial dysfunction are commonly implicated in cellular models of neurodegeneration [60,61]; however, the present study did not include a disease-related insult. Therefore, the observed changes in oxidative stress markers reflect the modulation of basal redox-related pathways rather than protection against induced oxidative damage. Likewise, although alterations in TAU phosphorylation are relevant to neurodegenerative diseases, the present findings do not demonstrate effects in a pathological context [62,63].

SH-SY5Y cells are a widely used model for studying intracellular signalling pathways relevant to neuronal biology. However, undifferentiated SH-SY5Y cells do not fully reproduce the phenotype of mature neurons, and therefore findings obtained in this model should be interpreted as cellular signalling responses rather than direct neuronal or in vivo effects. Additional studies in differentiated neuronal models and disease-relevant conditions would be required to further establish the functional implications of these findings.

In the present study, insulin modulated key intracellular signalling pathways in SH-SY5Y neuroblastoma cells under basal culture conditions. Insulin exerts important physiological functions in both peripheral tissues and the central nervous system. Therefore, the signalling effects observed in the study should be interpreted within the context of insulin’s pleiotropic biological actions. Although systemic insulin administration may influence glucose homeostasis, alternative strategies such as intranasal insulin delivery have been investigated to enhance central nervous system insulin signalling while minimizing peripheral metabolic effects. Furthermore, insulin activated the PI3K/AKT pathway, as evidenced by increased AKT phosphorylation and the inhibitory phosphorylation of GSK-3β at Ser9. These changes were associated with a reduction in tau phosphorylation at Ser396, suggesting the regulation of tau-related signalling downstream of insulin receptor activation. In parallel, insulin treatment was associated with changes in cellular redox homeostasis, including increased NRF2 protein expression, the upregulation of antioxidant enzymes (Cu/Zn-SOD and Mn-SOD), and reduced lipid peroxidation as reflected by decreased MDA levels. Together, these findings indicate that insulin influences signalling networks involved in cellular survival and oxidative stress regulation in this cellular model.

## 4. Materials and Methods

### 4.1. Cell Culture and Insulin Treatment

Human neuroblastoma SH-SY5Y cells (ATCC, Manassas, VA, USA) were cultured in Dulbecco’s Modified Eagle Medium (DMEM) (Gibco, Thermo Fisher Scientific, Waltham, MA, USA) supplemented with 10% fetal bovine serum (FBS) (Gibco, Thermo Fisher Scientific, Waltham, MA, USA), 1% penicillin–streptomycin, and 2 mM L-glutamine (Gibco, Thermo Fisher Scientific, Waltham, MA, USA) at 37 °C in a humidified incubator containing 5% CO_2_.

Cells were grown to approximately 70–80% confluence before experimental treatments. Prior to insulin exposure, cultures were incubated for 4 h in DMEM containing 1% FBS to reduce basal growth factor signalling. Cells were then treated with human recombinant insulin (Sigma-Aldrich, St. Louis, MO, USA) at a final concentration of 10^−8^ M (10 nM). The insulin concentration selected for this study (10^−8^ M) was based on previous studies using SH-SY5Y cells and other neuronal models, in which this concentration effectively activated insulin signalling pathways, including AKT and downstream targets, without evidence of cytotoxicity. The aim of the present study was to characterize the cellular signalling responses induced by insulin at a physiologically relevant and widely used concentration. We also acknowledge that response analyses could provide additional information.

Treatment times were selected according to the experimental endpoint:

- AKT and GSK-3β phosphorylation: 30 min;
- NRF2 and pTAU expression: 24 h;
- MTT assay: 24 h;
- LDH assay: 24 h;
- Caspase-3 activity: 24 h;
- Cu/Zn-SOD and Mn-SOD expression: 24 h.

Incubation times were selected according to the temporal dynamics of insulin signalling. Short-term exposure (30 min) was used to evaluate rapid phosphorylation events, including AKT and GSK-3β activation, whereas longer incubation periods (24 h) were employed to assess downstream cellular responses, including tau phosphorylation, oxidative stress-related markers, and cell survival parameters.

### 4.2. MTT Assay

Cell metabolic activity was assessed using the MTT reduction assay. SH-SY5Y cells were seeded into 96-well plates and treated with insulin (10^−8^ M) for 24 h. Following treatment, the culture medium was replaced with serum-free medium containing MTT (0.5 mg/mL in PBS), and cells were incubated for 4 h at 37 °C. After incubation, the medium was removed and the resulting formazan crystals were dissolved in dimethyl sulfoxide (DMSO). Absorbance was measured at 570 nm using a microplate reader. Results were expressed as a percentage of the control values.

### 4.3. Trypan Blue Exclusion Assay

Cell metabolic activity and viability were assessed using the Trypan Blue exclusion method. SH-SY5Y cells were seeded at a density of 7 × 10^4^ cells per 35 mm culture dish and allowed to attach overnight. Cells were then incubated in the absence (control) or presence of insulin (10^−8^ M) for 24 h. Following treatment, cells were harvested, mixed with 0.4% Trypan Blue solution, and counted using a hemocytometer. Viable cells were identified by exclusion of the dye and expressed as the total viable cell number.

### 4.4. Caspase-3 Activity Assay

Caspase-3 activity was determined using the ApoAlert^®^ CPP32/Caspase-3 Assay Kit (Clontech Laboratories, Palo Alto, CA, USA) following the manufacturer’s instructions.

Following treatment, SH-SY5Y cells were collected, washed twice with ice-cold PBS, and centrifuged. Cell pellets were stored at −80 °C until analysis. Samples were lysed, and equal amounts of protein were incubated with 50 μM Ac-DEVD-AFC substrate for 1 h at 37 °C. Fluorescence was measured using a microplate reader with excitation at 360 nm and emission at 530 nm.

### 4.5. Lactate Dehydrogenase (LDH) Assay

Cell membrane integrity was evaluated by measuring lactate dehydrogenase (LDH) (Thermo Fisher Scientific, Waltham, MA, USA) activity released into the culture medium.

After treatment, culture supernatants were collected and LDH activity was determined spectrophotometrically by monitoring NADH oxidation at 340 nm. Results were expressed relative to control values.

### 4.6. Lipid Peroxidation Assay

Lipid peroxidation was assessed by measuring malondialdehyde (MDA) levels using a commercially available Lipid Peroxidation (MDA) Assay Kit (Abcam, Cambridge, UK; Cat. No. ab118970).

SH-SY5Y cells were treated with insulin (10^−8^ M) for 24 h. Culture supernatants were collected and centrifuged at 1500× *g* for 10 min to remove debris. Samples were incubated with thiobarbituric acid (TBA) at 95 °C for 60 min according to the manufacturer’s protocol. Absorbance was measured at 532 nm using a microplate reader. MDA concentrations were calculated from an MDA standard curve and expressed relative to control values.

### 4.7. Western Blot Analysis

Cells were washed twice with ice-cold phosphate-buffered saline (PBS) and lysed in SDS sample buffer containing 0.125 M Tris-HCl (pH 6.8), 2% SDS, 0.5% β-mercaptoethanol, 1% bromophenol blue, and 19% glycerol. Protein concentrations were determined using the modified Lowry method.

Equal amounts of protein (20–40 μg) were separated by SDS-PAGE and transferred onto nitrocellulose membranes using standard procedures. Membranes were blocked with 5% non-fat dry milk in Tris-buffered saline containing 0.05% Tween-20 (TBS-T) for 1 h at room temperature and incubated overnight at 4 °C with the corresponding primary antibodies. Exposure times were adjusted to ensure signal acquisition within the linear range of detection. After washing with TBS-T, membranes were incubated for 1 h at room temperature with horseradish peroxidase-conjugated goat anti-mouse or goat anti-rabbit secondary antibodies. Immunoreactive bands were visualized using an enhanced chemiluminescence (ECL) detection system and quantified by densitometric analysis using Bio-Rad image analysis software (version 6.1 Bio-Rad Laboratories, Hercules, CA, USA). Protein expression levels were normalized to β-actin.

The following primary antibodies were used: AKT (Cat. No. GRW10110, GenoChem World, Bonrepòs i Mirambell, Valencia, Spain) (1:1000), phospho-AKT (Cat. No. 07-789, Millipore, Burlington, MA, USA) (1:1000), NRF2 (Cat. No. SAB5700720, Sigma-Aldrich, St. Louis, MO, USA) (1:1000), GSK-3β (Cat. No. GRW10122, GenoChem World, Bonrepòs i Mirambell, Valencia, Spain) (1:1000), phospho-GSK-3β (Ser9) (Cat. No. ab75814, Abcam, Cambridge, UK) (1:1000), total tau (Cat. No. GRW10158, GenoChem World, Bonrepòs i Mirambell, Valencia, Spain) (1:1000), phospho-tau (Ser396) (Cat. No. A34931, Antibodies.com, Cambridge, UK) (1:1000), Cu/Zn-SOD (copper/zinc superoxide dismutase) (Cat. No. SAB5200083, Sigma-Aldrich, St. Louis, MO, USA) (1:1000), Mn-SOD (manganese superoxide dismutase) (Cat. No. SAB2102261, Sigma-Aldrich, St. Louis, MO, USA) (1:1000), and β-actin (Cat. No. A2228, Sigma-Aldrich, St. Louis, MO, USA) (1:5000), Secondary antibodies included goat anti-mouse IgG (Cat. No. ab205719, Abcam, Cambridge, UK) (1:2000), or goat anti-rabbit IgG (Cat. No. ab97080, Abcam, Cambridge, UK) (1:2000).

### 4.8. Statistical Analysis

Statistical analyses were performed using GraphPad Prism (version 10). Data are presented as mean ± standard deviation (SD) of at least four independent biological replicates per group. The normality of data distribution was assessed using the Shapiro–Wilk test. For comparisons between two groups (control vs. insulin-treated cells), statistical significance was determined using an unpaired two-tailed Student’s *t*-test. A *p* value < 0.05 was considered statistically significant. Individual data points representing independent biological replicates are displayed in all quantification graphs to ensure the transparency and visualization of biological variability.

## Figures and Tables

**Table 1:** Effect of insulin on SH-SY5Y cell number and viability. Data are mean ± SD of independent experiments (*n* = 4). Data are expressed as percentage of control (100%). Control values were set to 100%. Statistical analysis was performed using Student’s *t*-test (* *p* < 0.05 vs. control).

	Seeding Cells (10^4^/35 mm Dish)	5 Days of Culture	24 h Treatment	% Viable Cell Number
**Control**	7	11.65 ± 0.22	11.75 ± 0.24	100
**Ins**	7	11.56 ± 0.32	13.77 ± 0.36	119.15 *

## Data Availability

The original contributions presented in this study are included in the article. Further inquiries can be directed to the corresponding author.
